# Long Non-Coding RNAs (lncRNAs) in Heart Failure: A Comprehensive Review

**DOI:** 10.3390/ncrna10010003

**Published:** 2023-12-28

**Authors:** Shambhavi Jha, Vasanth Kanth Thasma Loganathbabu, Kasinathan Kumaran, Gopinath Krishnasamy, Kandasamy Nagarajan Aruljothi

**Affiliations:** 1 Department of Genetic Engineering, College of Engineering and Technology, SRM Institute of Science and Technology, Kattankulathur Campus, Chengalpattu 603203, Tamilnadu, India; sj7888@srmist.edu.in (S.J.); vt7848@srmist.edu.in (V.K.T.L.); kk3009@srmist.edu.in (K.K.); 2 Institute of Biomedicine, University of Turku, FI-20520 Turku, Finland; gopinath.krishnasamy@utu.fi

**Keywords:** long non-coding RNAs (lncRNAs), heart failure, diagnostic markers, therapeutic targets, gene regulation, cardiovascular biomarkers

## Abstract

Heart failure (HF) is a widespread cardiovascular condition that poses significant risks to a wide spectrum of age groups and leads to terminal illness. Although our understanding of the underlying mechanisms of HF has improved, the available treatments still remain inadequate. Recently, long non-coding RNAs (lncRNAs) have emerged as crucial players in cardiac function, showing possibilities as potential targets for HF therapy. These versatile molecules interact with chromatin, proteins, RNA, and DNA, influencing gene regulation. Notable lncRNAs like Fendrr, Trpm3, and Scarb2 have demonstrated therapeutic potential in HF cases. Additionally, utilizing lncRNAs to forecast survival rates in HF patients and distinguish various cardiac remodeling conditions holds great promise, offering significant benefits in managing cardiovascular disease and addressing its far-reaching societal and economic impacts. This underscores the pivotal role of lncRNAs in the context of HF research and treatment.

## 1. Introduction

Heart failure (HF) is a complex and impairing cardiovascular disease that affects millions of individuals worldwide, causing increasing rates of morbidity and mortality [1]. The molecular mechanisms underlying HF pathogenesis are multifaceted and involve the dysregulation of various genes and signaling pathways. Recent research has shed light on the crucial role of lncRNAs in modulating gene expression and cellular processes, making them prominent players in the context of HF [2,3].

Long non-coding RNAs (lncRNAs) constitute a diverse group of RNA molecules with lengths surpassing 200 nucleotides and lacking protein-coding abilities [4]. Initially disregarded as mere transcriptional background noise, lncRNAs have now emerged as pivotal regulators of gene expression and cellular processes. They exercise their impact via various mechanisms, including chromatin remodeling, transcriptional interference, and post-transcriptional changes [5,6,7]. Their involvement in regulating essential pathways within cardiovascular biology, such as apoptosis, inflammation, and the remodeling of cardiac tissues, are also highly noteworthy [8,9].

HF is characterized by the heart’s inability to properly pump blood, resulting in inadequate delivery to essential organs. Understanding the pathogenesis of HF necessitates recognizing the multifactorial aspects of the condition, which include modifications in heart structure and function, neurohormonal activation, and inflammatory responses [10,11]. HF progression commonly features cardiomyocyte hypertrophy, fibrosis, diminished contractility, and maladaptive cardiac remodelins [12,13]. Additionally, reduced cardiac output triggers the activation of neurohormonal factors such as the renin–angiotensin–aldosterone system (RAAS) and the sympathetic nervous system (SNS). This leads to vasoconstriction and retention of sodium and water, thereby enhancing HF. Thus, recognizing the complex interaction of these pathological processes is a prerequisite for the formulation of effective prevention strategies, early diagnosis, and management [14,15]. The association between lncRNAs and HF is a dynamic and rapidly evolving area of research. Increasing evidence suggests that disrupted lncRNAs play a role in the development and progression of heart failure by influencing processes such as cardiomyocyte hypertrophy, fibrosis, and contractility in multiple ways, which are discussed in much depth in this review [16,17].

Few lncRNAs have demonstrated their potential as diagnostic and prognostic markers, providing promising avenues for personalized treatment of HF [18,19]. This current understanding opens the door to further insights into the complex interactions between ncRNAs and their interconnected networks, revealing potential targets for treatment and diagnostic tools for this serious disease [20,21]. In the following sections, we will delve into specific lncRNAs linked with HF, understanding their regulatory mechanisms and their roles in crucial HF progression processes via various studies focusing on this area of research [22]. The potential therapeutic approaches targeting lncRNAs and the inherent challenges in implementing such knowledge in clinical practice will also be discussed. At its best, this review intends to emphasize the transformative ability of lncRNAs as potential keys to unravel the complexities of HF and enhance patient outcomes.

## 2. Understanding Heart Failure: A Brief Overview

HF is a complex cardiovascular disorder where the heart struggles to pump blood efficiently, leading to inadequate supply to vital organs [11,23]. It is essential to grasp the complexities of HF for effectively managing this condition, given its significant impact on clinical outcomes (Figure S1). To truly understand how HF develops, we need to consider its multifaceted nature. This involves changes in both the structure and function of the heart, as well as the activation of certain hormonal pathways and inflammatory response.

As HF progresses, we typically see enlargement of heart cells, the development of fibrous tissue, compromised heart muscle contractility, and the heart undergoing maladaptive changes. Additionally, hormonal responses like the renin–angiotensin–aldosterone system (RAAS) and the sympathetic nervous system (SNS) kick in due to reduced heart output, leading to blood vessel constriction and the retention of sodium and water. These factors further worsen HF. Therefore, gaining a deep understanding of how these various processes interact is essential for creating effective approaches for preventing HF, diagnosing it early, and providing proper management [24,25].

**Figure 1:**
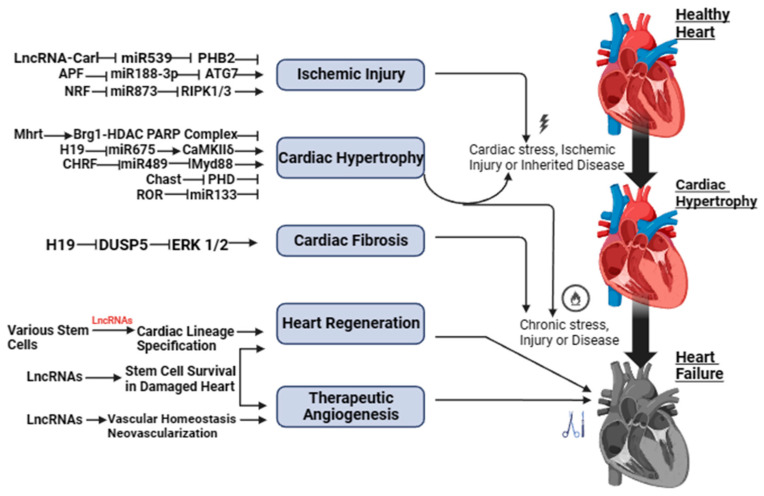
LncRNAs as possible therapeutic targets for heart failure.

Both Figure 1 and Figure S1 show a schematic illustration of some studies in vitro and in vivo on the study of regulatory activity of lncRNAs in HF. The activity of lncRNAs in HF is based on a fine line of regulation of complex molecular networks at the level of epigenetic changes, transcription, and post-transcriptional transformations, such as lncRNA/microRNA (miRNA)/mRNA connections, which are necessary for the control of proliferation and migration of cardiomyocytes, collagen synthesis, inflammation, atrial electrical remodeling, cardiac autonomic nervous function, and oxidative stress.

## 3. Exploring the Connection between lncRNA and Heart Failure

Lately, the complex connection between lncRNAs and HF has become a focal point in cardiovascular research. This relationship holds the promise of unveiling novel insights into the molecular underpinnings of HF and, consequently, transforming our approach to diagnosis and treatment of this prevalent and debilitating condition [4,26,27].

Transpiring studies have brought to light the pivotal roles played by lncRNAs in the pathogenesis of HF [19]. Greco et al. [17] demonstrated that lncRNAs are intimately involved in orchestrating processes such as cardiomyocyte hypertrophy, fibrosis, apoptosis, and contractility, collectively contributing to the development and progression of HF [28]. These regulatory molecules, once dismissed as transcriptional noise, are now recognized as central players in the complex web of molecular interactions driving HF pathophysiology. The ever-evolving high-throughput RNA sequencing technologies have allowed researchers to identify dysregulated lncRNAs in HF, both in animal models and in humans [29,30]. All of these findings propose that lncRNAs play an explicative role in the pathogenesis of, and may pose as a potential therapeutic target, for this life-threatening condition [31,32]. One of the remarkable aspects of the lncRNA–HF connection is the diversity of regulatory mechanisms involved [22,33]; for example, the lncRNA H19 has been shown to promote myocardial fibrosis by influencing the deposition of extracellular matrix components in the heart. H19 is a long non-coding RNA that has been associated with various biological processes, including cellular growth, differentiation, and apoptosis. Several articles suggest that lncRNA-H19 is a potent regulator of the progression of cardiac hypertrophy. They indicate that lncRNA-H19 is involved in the pathophysiological processes of cardiac hypertrophy, including calcium regulation, fibrosis, apoptosis, angiogenesis, inflammation, and methylation [34,35]. In contrast, the lncRNA Mhrt (Myosin Heavy Chain Associated RNA Transcripts) has been found to protect against HF by inhibiting cardiac hypertrophy [36]. These findings underscore the complex and context-dependent roles of lncRNAs in HF.

### Clinical Evidence

Recent clinical evidence shows that particular lncRNAs in HF can diagnose and give prognosis, possibly changing risk assessment and patient care. Li et al. and Kumarswamy et al. [37,38] identified lncRNAs, including ANRIL (Antisense Non-coding RNA in the INK4 Locus) and LIPCAR (mitochondrial long non-coding RNA uc022bqs.1), as prospective biomarkers for HF [39] due to their altered expression patterns in HF patients and their correlation with disease severity and prognosis. Their diagnostic and prognostic capabilities enable more accurate risk stratification and early HF identification, improving patient care [40].

The quantity and cellular origins of circulating lncRNAs in HF and their association with cardiac regeneration need more study [31,33]. The preliminary findings in this scholarly review shed light on lncRNAs and HF. The preliminary data imply that lncRNAs are increasingly relevant in coronary heart disease, heart failure, and hypertension [41]. Due to their modest number and unknown cell source, circulating lncRNAs are difficult to detect. The link between circulating lncRNAs and cardiac regeneration is unclear, reducing the sensitivity and specificity of employing them as heart failure diagnostic markers or therapeutic targets [31,42].

Therefore, we must acknowledge the uncertainties and challenges in lncRNA research, which is crucial for a comprehensive understanding. It is evident that while lncRNAs hold immense potential in deciphering heart-related conditions, navigating through these complexities demands rigorous evaluation and standardized methodologies. Emphasizing the need for stringent evaluation and standardized methodologies is pivotal. Because of their complexity, lncRNAs must be thoroughly investigated and their roles and interactions must be validated using strong methodologies [33]. Standardization across research methodologies and data analysis frameworks would enhance comparability and reliability among studies, fostering a more cohesive understanding of lncRNA functions in heart failure and related conditions [41]. This rigorous approach is essential for establishing concrete correlations between specific lncRNAs and their functional implications in heart failure. It will also ensure that findings are reproducible and applicable in clinical settings [31]. Moreover, lncRNA-targeted therapeutics must be systematically examined in preclinical and clinical studies, and the possible off-target effects and long-term impacts of modifying lncRNAs should be carefully considered at every stage [43,44].

## 4. Unraveling the Role of lncRNAs in Heart Disease Pathogenesis

According to present-day knowledge, lncRNAs have emerged as crucial players in various biological processes, including the development and maintenance of heart function [45,46]. Through extensive research and advances in RNA sequencing technology, it has been discovered that LncRNAs exhibit distinct expression patterns in human heart failure compared with normal donor hearts [29,47]. Hence, it could be said that LncRNAs may play a regulatory role in the pathogenesis of heart failure [26,48]. Several studies have also reported the detection of lncRNAs in plasma or urine samples, which indicates their potential as diagnostic markers for HF [49].

In the context of HF, lncRNAs have been linked to critical cellular processes such as cardiomyocyte hypertrophy, fibrosis, apoptosis, and contractility [17,44]. These regulatory roles of lncRNAs have important implications for the development and progression of HF [50]. For example, antisense lncRNA Kcna2 (Long non-coding RNA Potassium Voltage-Gated Channel Subfamily A Member 2) is linked to a higher prevalence of ventricular arrhythmias, which are irregular heartbeats that only impact the heart’s lower chambers in heart failure patients [51]. The study found that Kcna2 knockdown in the heart decreased the slow component of the rectifier potassium current (IKs) and prolonged action potentials in cardiomyocytes, consistent with the changes observed in heart failure. Conversely, Kcna2 overexpression in the heart significantly attenuated the CHF-induced decreases in the IKs, AP prolongation, and ventricular arrhythmias. This discovery highlights the role of lncRNAs in cardiac electrical remodeling and the development of arrhythmias in heart failure [51]. Additionally, lncRNA UCA1 (Long non-coding RNA urothelial carcinoma-associated 1) has been shown to promote the progression of cardiac hypertrophy, a condition commonly associated with various cardiovascular diseases such as heart failure. Cardiac hypertrophy is closely associated with a series of cardiovascular diseases, including heart failure and sudden death in particular. Therefore, understanding the pathogenesis of cardiac hypertrophy is crucial for improving its diagnosis and therapy. According to a study, lncRNA UCA1 is a novel regulator in cardiomyocyte hypertrophy through targeting the miR-184/HOXA9 axis [52]. The study found that lncRNA UCA1 was highly expressed in mice heart treated with transverse aortic constriction (TAC) and the cardiomyocytes treated with phenylephrine (PE). On the contrary, miR-184 was downregulated under the same conditions. The knockdown of UCA1 or the overexpression of miR-184 lessened the enlarged surface area of cardiomyocytes and the elevated expressions of fetal genes (ANP and BNP) induced by PE. Later, it was determined that miR-184 was a direct target of UCA1, whereas the mRNA HOXA9 was a target of miR-184. Rescue assays indicated that UCA1 promoted the progression of cardiac hypertrophy through competitively binding with miR-184 to enhance the expression of HOXA9 [52].

Moreover, lncRNA GAS5 (Long non-coding RNA Growth Arrest-Specific 5), which is known as a tumor suppressor gene, has been implicated in heart failure caused by myocardial infarction. GAS5 is a long non-coding RNA that is a member of the 5′ terminal oligo-pyrimidine class of genes. It is a small nucleolar RNA host gene, containing multiple C/D box snoRNA genes in its introns. The secondary RNA structure of the encoded transcript mimics glucocorticoid response element (GRE), which means it can bind to the DNA binding domain of the glucocorticoid receptor (GR) and block its activation, thereby stopping it from regulating the transcription of its target genes [51]. GAS5 has been linked to apoptosis and cellular growth arrest. It may also function as a tumor suppressor; in several tissues, its downregulation has been linked to cancer. Several lncRNAs, including GAS5, are crucial regulators of cell differentiation, development, and disease, and their expression is often associated with stress conditions in the heart. The dysregulation of lncRNAs is associated with a variety of cardiac diseases, including heart failure [53]. However, the specific role of GAS5 in heart failure is yet to be determined. To study the same, a study was conducted by Wang and Xie [51] that aimed to evaluate the importance and its role in heart failure development post-myocardial infarction of lncRNA GAS5 in hypoxia-injured H9c2 cells, which is a type of cardiac muscle cell line that was derived from embryonic rat hearts. The results of this study demonstrated that lncRNA GAS5 was significantly upregulated in hypoxia-injured H9c2 cells and its expression inhibited the progression of heart failure caused by myocardial infarction [51]. It is also known that LncRNAs take part in interactions with several major regulators that are involved in the modulation of calcium ions, such as zinc finger antisense 1 (ZFAS1), myocardial infarction-associated transcript (Miat), and zinc finger protein antisense RNA (ZNF593-AS) [54,55]. Theoretically, the interaction between ZFAS1 and SERCA2a (Sarco/Endoplasmic Reticulum Calcium ATPase 2a, a crucial protein found in the sarcoplasmic reticulum membranes of cardiac muscle cells that controls calcium ion transport and heart muscle contraction and relaxation) leads to an excessive buildup of intracellular calcium ions, which causes abnormal calcium ion fluctuations in cardiomyocytes and impairs their ability to contract effectively. Miat has been observed to interfere with the pan-RNA splicing process, resulting in a reduction in the expression levels of SERCA2a and RyR2 (Ryanodine Receptor 2, a calcium channel mostly present in the cardiac muscle cells’ sarcoplasmic reticulum that is crucial for regulating the release of calcium during muscle contraction, particularly in the heart) [54,55]. Consequently, this disruption contributes to compromised contractility.

In addition to that, in a study conducted by Jiao et al., it was observed that lncRNA-ZFAS1 triggers mitochondria-mediated apoptosis in mice with myocardial infarction by inducing an excessive accumulation of cytosolic Ca^2+^ [56]. The reversible nature of the effect of ZFAS1 was observed following the knockdown, suggesting that targeting ZFAS1 with anti-ZFAS1 agents could serve as a novel therapeutic approach to safeguard cardiomyocytes from apoptosis generated by myocardial infarction [57,58]. Additionally, the LncRNA Caren (cardiomyocyte-enriched non-coding transcript), which is highly expressed in cardiomyocytes, plays a vital role in preserving cardiac function when pressure is high. It achieves this by suppressing the ataxia telangiectasia mutated (ATM)/DNA damage response (DDR) pathway and modulating the deacetylase activity of sirtuin 2 [59]. Another lncRNA, known as H19, shows a high degree of conservation and serves as a critical factor in the early stages of postnatal development as well as in the pathogenesis of several disorders [9,60,61]. Similarly, the expression of LncKCND1 (potassium voltage-gated channel subfamily D member 1) is increased in models of heart failure, and it functions to suppress the enlargement of cardiomyocytes by the production of miR-675 [62]. This microRNA specifically targets CaMKIIδ (Calcium/Calmodulin-Dependent Protein Kinase II delta, an enzyme that plays an important part in a variety of physiological activities, including calcium control and the formation of memory in neurons, as well as heart muscle contraction). Other lncRNAs involved in epigenetic regulation, such as the cardiac-hypertrophy-associated epigenetic regulator (CHAER), have direct interaction with PRC2 (Polycomb Repressive Complex 2, a protein complex involved in gene regulation control) [63]. This interaction does not allow PRC2 to target certain genomic areas, leading to a decrease in H3K27me3 (Histone 3 Lysine 27 trimethylation, a specific epigenetic modification that is associated with gene repression and in the regulation of chromatin structure and gene expression) levels at the promoter regions of genes implicated in cardiac hypertrophy [64].

LncRNAs have a substantial influence on the progression of cardiac fibrosis and heart failure due to myocardial infarction [65]. These molecules possess the ability to regulate the expression of neighboring genes, promote the growth and viability of fibroblasts, and impede the interaction between COTL1 (Coactosin-like protein 1, a protein involved in the regulation of actin filament dynamics) and TRAP1 (TNF receptor-associated protein 1, a member of the HSP 90- Heat Shock Protein 90 family and is found majorly in the mitochondria), allowing the transdifferentiation of myofibroblasts into cardiac fibroblasts [66].

Some exceptional work conducted by Han et al. investigated the expression patterns of lncRNA in human cardiac fibroblasts (HCFs) affected by cardiac fibrosis. The results of the functional analysis demonstrated that a total of 176 lncRNAs were elevated, while 526 lncRNAs were downregulated in human dermal fibroblasts (HCFs) that were stimulated with transforming growth factor-beta (TGF-β). The identified target genes were shown to be associated with several biological processes, including focal adhesion, metabolic pathways, the Hippo signaling system, the PI3K-Akt (Phosphoinositide 3-kinase-Protein Kinase B) signaling pathway, control of actin cytoskeleton, and hypertrophic cardiomyopathy [28,67,68]. The novel lncRNAs identified as NONHSAG005537 and NONHSAG017620 were also found to exert inhibitory effects on the proliferation, migration, invasion, and transformation of human cardiac fibroblasts (HCFs).

The CANTOS (Canakinumab Anti-inflammatory Thrombosis Outcome Study) trial also provides evidence supporting the potential efficacy of anticytokine therapy as a strategy for enhancing heart function in patients with myocardial infarction. Nevertheless, there is a significant lack of understanding regarding the specific mechanisms by which lncRNAs contribute to the development of cardiac dysfunction. However, according to a recent study, it has been discovered that the initiation of heart failure is facilitated by the action of SOX2-OT (SOX2 Overlapping Transcript, an lncRNA encoding factors that are essential to embryonic development and also involved in cell fate determination) through its interaction with miR-455-3p, increasing the expression of TNF receptor-associated factor 6 (TRAF6) and the subsequent activation of the NF-κb (Nuclear Factor kappa-light-chain-enhancer of activated B cells, a protein complex that regulates the expression of immune response genes) signaling pathway [69,70]. A translational study was conducted to investigate the role of lncRNAs in the antiviral response of individuals with coxsackievirus-B3 cardiomyopathy as well. This investigation revealed that the lncRNA MALAT1 (Metastasis-Associated Lung Adenocarcinoma Transcript 1), together with its enzymatic processing product called MALAT1-associated short cytoplasmic RNA, plays a significant role in providing favorable immunoregulatory capabilities in this context [71,72]. Increased levels of glucose are found to stimulate the expression of myocardial infarction-associated transcript (MIAT) and increase the expression of DAPK2 (Death-Associated Protein Kinase 2), promoting apoptosis in cardiomyocytes [73,74,75]. Furthermore, emerging evidence suggests that lncRNAs are involved in inflammation, oxidative stress, and angiogenesis, all of which play critical roles in HF pathophysiology [76]. Understanding the exact roles of these lncRNAs and their interactions with coding genes is important for understanding the molecular mechanisms of [77].

## 5. Recent Studies on lncRNA and Heart Failure

Recently, there has been a surge in research delving into the connection between lncRNAs and HF, leading to significant strides in our grasp of the molecular workings behind this complex cardiovascular condition [78]. These studies, marked by their depth and sophistication, have illuminated the pivotal roles played by specific lncRNAs in heart dysfunction development, offering insights into both the underlying biology and potential clinical applications [78]. A standout study by Wang et al. [34] spotlighted the crucial role of the lncRNA H19 in regulating cardiac fibrosis, a hallmark of HF. Their findings demonstrated that H19 acts as a “competing endogenous RNA” (ceRNA), essentially acting as a sponge for miR-675-5p. This leads to an upregulation of connective tissue growth factor (CTGF), which in turn promotes cardiac fibrosis. This discovery provides a key link between lncRNAs and the adverse cardiac remodeling seen in HF [79].

In addition, a recent clinical study by Zhang et al. [48] homed in on the lncRNA CHAST (Cardiac Hypertrophy-Associated Transcript) as a potential biomarker for HF. Their research unveiled that CHAST is significantly elevated in HF patients and correlates with the severity of the condition and the likelihood of adverse cardiac events. This underscores the diagnostic and prognostic potential of lncRNAs, emphasizing their value in assessing risk and managing patient care. Researchers have also made headway in detecting lncRNAs in plasma or urine, noting dynamic changes during the onset and progression of heart failure, as mentioned before [49,80]. Some specific lncRNAs, like “long intergenic non-coding RNA predicting cardiac remodeling” and “myocardial infarction-associated transcript”, have shown promise as potential diagnostic markers for HF. Additionally, unfolding studies on lncRNA GAS5 have shed light on its role in regulating apoptosis, autophagy, and inflammation in the context of heart failure [51]. Table 1 highlights some of the key findings on the implications of lncRNA on HF.

Furthermore, investigations have hinted at the involvement of lncRNAs in coronary heart disease, heart failure, and hypertension, indicating their potential impact on a broad spectrum of cardiovascular conditions [31]. High-throughput RNA sequencing technologies have been instrumental in uncovering the dysregulation of lncRNAs in heart failure, potentially paving the way for diagnostic breakthroughs and future therapeutic targets in several studies that have been discussed previously [21]. However, it is essential to recognize the complexity of the lncRNA–HF relationship. The multifaceted roles of lncRNAs in various cellular processes, including cardiomyocyte hypertrophy, apoptosis, and inflammation, suggest that their influence on HF is far-reaching and context-dependent [81]. Bringing these research findings into clinical practice also calls for tackling certain challenges like standardizing methodologies for lncRNA analysis, which needs robust validation across diverse patient populations, and developing noninvasive techniques for lncRNA detection, which are key areas that require ongoing exploration and refinement [53].

## 6. Insights and Interpretations of the Study

Recent research investigating the relationship between lncRNAs and HF has unveiled a rich tapestry of molecular mechanisms with profound implications for our understanding of heart health [8]. These findings, characterized by their depth and clinical relevance, offer both mechanistic insights and potential clinical applications. A study by Wang et al. [34] stands out as a significant contribution to our understanding of lncRNAs in HF. They revealed the pivotal role of the lncRNA H19 in cardiac fibrosis, a hallmark of HF. This study elucidated the mechanisms by which H19 acts as a competing endogenous RNA (ceRNA) to sponge miR-675-5p, leading to the upregulation of connective tissue growth factor (CTGF) and promoting cardiac fibrosis. This complex molecular interplay between lncRNAs, microRNAs, and protein-coding genes highlights the complications of lncRNA-mediated regulation in HF pathogenesis. Another important study by Zhang et al. [48] demonstrated the clinical utility of lncRNAs as biomarkers in HF. They identified the lncRNA CHAST as significantly upregulated in HF patients and correlated its expression levels with disease severity and adverse cardiac events. This finding underscored the diagnostic and prognostic potential of lncRNAs, offering clinicians a novel tool for risk assessment and patient management in HF. Additionally, preclinical studies targeting lncRNAs such as MALAT1 and NEAT1 (Nuclear-Enriched Abundant Transcript 1, a nucleus-based long non-coding RNA (lncRNA) that creates and maintains paraspeckles; it regulates gene expression and cellular behaviors including stress and growth) have demonstrated the ability to attenuate HF and improve cardiac function through this study [28,71,72]. However, the multifaceted roles of lncRNAs in HF extend beyond a single HF condition like fibrosis. Other studies, such as those conducted by Thum et al. [82], Tao et al. [81], and Devaux et al. [9], have revealed the involvement of lncRNAs in processes such as cardiomyocyte hypertrophy, apoptosis, inflammation, and extracellular matrix dynamics [64,83].

The collective results of these initial observations indicate that lncRNAs exhibit considerable potential as diagnostic indicators and innovative targets for cardiovascular conditions [21,31]. Translating these research findings into clinical practice presents challenges that demand attention. As previously mentioned several times, standardized methodologies for lncRNA analysis, robust validation across diverse patient populations, and the development of noninvasive techniques for lncRNA detection are crucial steps in harnessing the diagnostic and therapeutic potential of lncRNAs in heart health [53,65,84]. There are many other challenges involved, such as the origin of these circulating lncRNAs remaining uncertain and their association with heart regeneration not being comprehensively elucidated so far. These aforementioned restrictions have a substantial impact on the sensitivity and specificity of utilizing lncRNAs as diagnostic indicators and therapeutic targets in the context of cardiovascular disorders. Therefore, interpreting the research findings on the impact of lncRNAs on heart health is an ongoing endeavor with the potential to revolutionize our approach to HF diagnosis, treatment, and management.

## 7. Implications for Future Medical Research and Treatments

The emerging body of research concerning lncRNAs within the framework of HF not only contributes to the advancement of our comprehension of HF pathophysiology but also exhibits significant potential for forthcoming medical investigations and innovative therapeutic approaches [82,85]. The outcomes of current studies have profound effects that will impact the field of cardiovascular medicine and therapeutic intervention [86].

Firstly, the identification of dysregulated lncRNAs in HF opens up avenues for targeted therapeutic interventions [25]. As our knowledge of specific lncRNAs involved in HF deepens, it becomes possible to design novel therapeutic strategies that aim to modulate these lncRNAs. In other words, the modulation of lncRNA expression levels or manipulation of lncRNA activity has the potential to ameliorate detrimental cardiac remodeling, fibrosis, and hypertrophy. For example, the development of small molecules or gene therapies that selectively target and modify the expression of specific lncRNAs could offer promising treatment options for HF [17,76]. The investigation of the molecular functions of lncRNAs, specifically H19 [34] and Mhrt [36], in the pathophysiology of HF presents great potential avenues for the development of targeted therapeutic interventions [87,88], which implies and lays more emphasis on the aim to investigate the complex aspects of the advancement of secure and efficient therapies based on long non-coding RNA (lncRNA), maybe employing innovative methods of delivery.

Secondly, the potential diagnostic and prognostic value of lncRNAs in HF presents an opportunity to refine patient management strategies. The integration of lncRNA profiles into clinical practice may enhance risk stratification, early diagnosis, and personalized treatment approaches. This could lead to a more effective allocation of resources and improved patient outcomes [38,65]. For example, the utilization of distinct lncRNAs, namely, CHAST [48] and ANRIL, as diagnostic biomarkers presents amazing opportunities for precision diagnostics in the context of HF. These biomarkers have the potential to improve risk assessment, facilitate early diagnosis, and offer more precise prognostic information. Therefore, it is important to prioritize the advancement of noninvasive diagnostic instruments, such as blood-based assays, with the aim of streamlining their integration into ordinary clinical practice.

Thirdly, exploring the dynamic regulation of lncRNAs in response to HF therapies could provide insights into treatment responsiveness and guide treatment amendments [89]. Understanding how lncRNA expression profiles change with different interventions may help identify optimal therapeutic strategies for individual patients, moving us closer to personalized medicine in HF management [90,91]. The diverse functions of lncRNAs in HF can be used to adopt a tailored therapy strategy resulting in the most favorable outcomes. In fact, the optimization of therapeutic effectiveness and the reduction of undesirable effects can also be achieved by tailoring treatment regimens according to the unique lncRNA profiles of individual patients. Moreover, the use of combinatorial strategies that concurrently address several lncRNAs or integrate lncRNA-targeted therapies with established treatments for HF has the potential to augment therapeutic effectiveness in the bigger picture [35,92].

Notably, the assessment of safety and specificity is of the utmost importance as the development of lncRNA-targeted treatments progresses. In order to ensure the safety of experimental treatments, it is imperative that both preclinical and clinical trials thoroughly evaluate potential off-target effects and long-term repercussions [46,93]. The rapid progress of technology, shown by the emergence of single-cell sequencing and CRISPR-based genome editing, has the potential to expedite the investigation of lncRNA and the creation of therapeutic interventions in a safer and more specific manner [94]. These techniques have the capability to offer a more profound understanding of lncRNA functionality and facilitate accurate modulation of lncRNA expression.

Finally, translational research plays a crucial role in bridging the divide between fundamental scientific inquiry and practical clinical implementation. The diagnostic capacity of lncRNAs presents the possibility of identifying HF at an early stage with accuracy, while tailored therapeutic approaches show potential in managing the advancement of HF [21,95]. The integration of personalized medicine, in-depth mechanistic understanding, and the utilization of combination treatments have the potential to significantly augment therapeutic techniques. Anyway, it is imperative to acknowledge and prioritize safety considerations while also embracing technical progress in order to effectively exploit the promise of lncRNAs in the treatment and control of heart failure. Therefore, the establishment of collaborations among researchers in the fields of basic science, clinical medicine, and industry is extremely crucial in order to effectively transfer these promising findings related to long non-coding RNA (lncRNA) into practical clinical therapies.

Hence, as the body of research in this particular domain continues to advance, it becomes increasingly apparent that lncRNAs will assume a crucial role in influencing the trajectory of cardiovascular medicine in the next years.

## 8. Conclusions

In a nutshell, recognizing the role of lncRNA in managing HF marks a significant shift in how we perceive this complex heart condition. These lncRNAs play a crucial role in processes like heart cell growth, fibrosis, cell death, and contraction strength, revealing fresh insights into what triggers HF. Moreover, certain lncRNAs show promise in diagnosing and predicting HF, opening doors for better personalizing patient care and spotting HF early on. Exploring treatments that target lncRNAs could address the core issues driving HF and expand our treatment options.

Still, it is vital to acknowledge that working with lncRNAs brings its own set of challenges and complexities. We need consistent methods, thorough validation, and a clear understanding of how safe and specific lncRNA-based treatments are before we can place them into practice. Yet, as we learn more about lncRNAs in HF, the potential for groundbreaking advances in patient care grows. Tapping into the potential of lncRNAs as tools for diagnosis and treatment could transform how we approach managing HF, offering new hope for both patients and their healthcare teams. In the future, research should focus on untangling the complex web of lncRNA interactions and developing safe, effective strategies for applying them in clinical settings, ultimately enhancing the outlook and quality of life for those dealing with heart failure.

## Figures and Tables

**Table 1:** Recent key findings on LncRNAs and their association with heart failure.

Key Findings in Recent Times	Implications for HF
NEAT1 promotes cardiac hypertrophy and fibrosis through miR-140-5p inhibition [37].	The potential therapeutic target for cardiac remodeling in HF.
Identified dysregulated lncRNA-mRNA networks in HF with preserved ejection fraction (HFpEF) [77].	Understanding HfpEF pathophysiology through lncRNA-mRNA interactions.
MALAT1 drives inflammation in HF through NF-κB activation [27].	Insights into inflammation-mediated HF progression.
HRTCT1 is elevated in HF patients and correlates with disease severity and adverse outcomes [74].	Promising diagnostic and prognostic markers for HF.
H19 contributes to cardiac fibrosis by sponging miR-675-5p, upregulating CTGF [73].	Potential therapeutic target for fibrosis in HF.
CHAST is upregulated in HF patients, and correlated with disease severity and clinical outcomes [48].	Diagnostic and prognostic biomarkers for HF.
Specific lncRNA signatures in peripheral blood can discriminate HF patients from controls [37].	Noninvasive tool for early HF diagnosis and risk assessment.
Wisper influences cardiac fibrosis via focal adhesion regulation [44].	Potential target to mitigate fibrosis in HF.
Discovered lncRNA KCND1 influencing hypertrophy-associated gene expression in cardiomyocytes. Enhanced hypertrophic response in HF models [62].	Insights into molecular pathways contributing to cardiomyocyte hypertrophy in HF. Potential therapeutic targets to prevent adverse remodeling.
Demonstrated GAS5 silencing’s protective role against hypoxia-induced cardiomyocyte injury through miR-21/PTEN pathway regulation [51].	Potential therapeutic avenue for attenuating hypoxia-induced cardiac injury in HF.

## Supplementary Materials

The following supporting information can be downloaded at: https://www.mdpi.com/article/10.3390/ncrna10010003/s1, Figure S1: A general list of symptoms localized to specific organs and regions that are an indication of heart failure.
