# Supplementary material for: Long Non-Coding RNAs (lncRNAs) in Heart Failure: A Comprehensive Review
Source: Noncoding RNA. 2023 Dec 28;10(1):3. doi: 10.3390/ncrna10010003 (PMC10801533; doi:10.3390/ncrna10010003)
Supplement: Supplementary file 1 [file ncrna-10-00003-s001.zip › ncrna-2789134-supplementary.pdf]

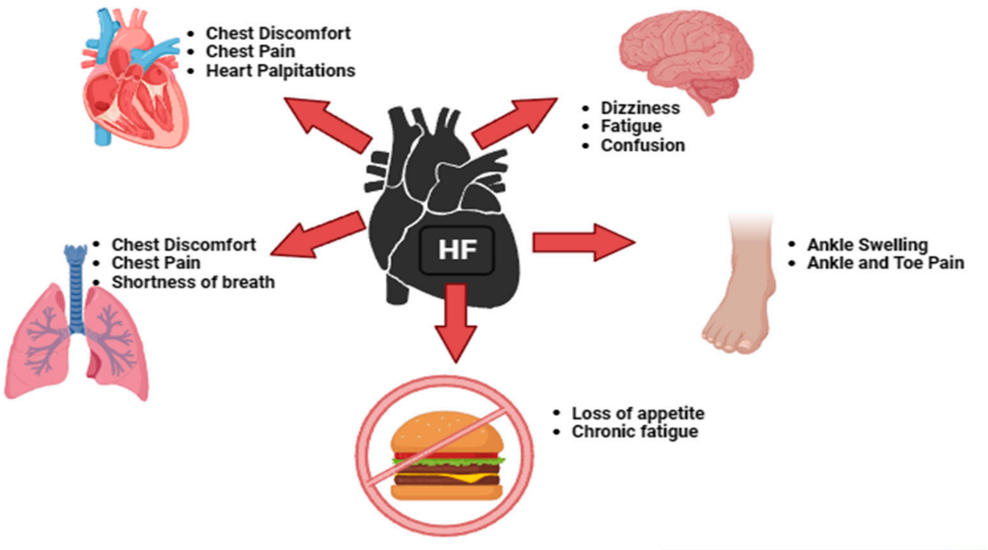

Figure S1. A general list of symptoms localized to specific organs and regions that are an indication of heart failure.
